# Theoretical Study on Reactions of Triplet Excited State Thioxanthone with Indole

**DOI:** 10.3390/ijms10104284

**Published:** 2009-11-20

**Authors:** Liang Shen, Hong-Fang Ji

**Affiliations:** Shandong Provincial Research Center for Bioinformatic Engineering and Technique, Center for Advanced Study, Shandong University of Technology, Zibo 255049, China; E-Mail: shen@sdut.edu.cn (L.S.)

**Keywords:** thioxanthone, indole, triplet excited state, deactivation, quantum chemical calculation

## Abstract

In the present work, a theoretical study on the deactivation of triplet excited (T_1_) state thioxanthone (TX) by indole (INH) was performed, based on density functional theory calculations. Three feasible pathways, namely direct electron transfer from INH to T_1_ state TX, electron transfer followed by proton transfer from INH^.+^ to TX^.−^, and H-atom transfer from nitrogen of INH to keto oxygen of T_1_ state TX, were proposed theoretically to be involved in T_1_ state TX deactivation by INH.

## 1. Introduction

Thioxanthone (TX) and its derivatives are efficient photosensitizers, which have attracted much attention in recent years owing to their broad spectrum of antitumor activities and great potential to be developed as novel antitumor agents [1–3]. It has been reported that photoexcited TX can cause DNA damage [4]. It is known that photosensitization involves two mechanisms, *i.e.,* direct reaction with substrates (*e.g.,* DNA, amino acids and proteins) (Type I) or damage via intermediacy of oxygen (through energy or electron transfer processes with molecular oxygen to generate toxic reactive oxygen species) (Type II). As the relatively long-lived triplet excited (T_1_) state is mainly responsible for the photosensitization reactions, exploring the deactivating processes of TX will be helpful to understand its photosensitization properties. The indole moiety exists in many bioorganic compounds like the amino acid tryptophan and in tryptophan-containing proteins. Therefore, in the present work, the energetics describing the deactivation of the T_1_ state TX by INH have been investigated using quantum chemical calculations.

## 2. Results and Discussion

Upon irradiation, ground (S_0_) state TX is initially excited to singlet excited (S_1_) state, which may then reside in the T_1_ state through intersystem crossing:

$$\text{TX}\;({\text{S}}_{0})\;\overset{h\upsilon }{\to }\;\text{TX}\;({\text{S}}_{1})\;\overset{ISC}{\to }\;\text{TX}\;({\text{T}}_{1})$$

According to the electronic parameters of TX and INH (Table 1), the feasibilities of the possible deactivating pathways of T_1_ state TX by INH may be examined theoretically.

First of all, T_1_ state TX may be deactivated by INH through a direct energy transfer process as represented in Equation 1:

(1) $$\text{TX}({\text{T}}_{1})+\text{INH}\to \text{TX}+\text{INH}({\text{T}}_{1})$$

Table 1 lists the TD-DFT estimated E_T1_ of TX and INH. The theoretical E_T1_ of TX (2.78 eV) and INH (3.26 eV) are close to the experimental values (2.84 eV for TX and 3.12 eV for INH) [5,6], which verifies the calculation methods. As INH possesses higher E_T1_ relative to TX, the direct energy transfer based deactivation pathway is unfeasible. Moreover, it is worth mentioning that as E_T1_ of TX is higher than the energy needed to bring ^3^O_2_ to singlet excited state (^1^O_2_), 1.05 eV ${(}^{3}\sum_{g}^{-}\to \Delta_{\text{g}}^{1})$ or 1.65 eV ${(}^{3}\sum_{g}^{-}\to^{1}\sum_{g}^{+})$, through direct energy transfer T_1_ state TX can photogenerate singlet oxygen (^1^O_2_), which may be involved in the DNA photooxidation by TX [4]:

(2) $$\text{TX}({\text{T}}_{1}){+}^{3}{\text{O}}_{2}\to \text{TX}{+}^{1}{\text{O}}_{2}$$

The second deactivating pathway is direct electron transfer between T_1_ state TX and INH (Equations 3 and 4).

(3) $$\text{TX}({\text{T}}_{1})+\text{INH}\to {\text{TX}}^{.-}+{\text{INH}}^{.+}$$

(4) $$\text{TX}({\text{T}}_{1})+\text{INH}\to {\text{TX}}^{.+}+{\text{INH}}^{.-}$$

Table 1 lists the theoretically estimated electronic parameters to characterize the molecular electron-donating or electron-withdrawing potentials for TX and INH. The feasibility of pathway (3) relies on the summation of AEA_T1_ of TX and AIP of INH, which is negative (Table 1). Thus, it can be inferred that the direct electron transfer from INH to T_1_ state TX is favorable. In contrast, the electron transfer from T_1_ state TX to INH (Equation 4) is theoretically unfeasible because of the positive value of total reaction energy (summation of AIP_T1_ of TX and AEA of INH). In previous study, the electron transfer-based DNA oxidation by photoexcited TX has been reported [4]. The electron transfer process has also been reported to be involved in the deactivation of T_1_ state TX by amines or indolic derivatives [7,8].

In addition, based on the experimentally identified formations of the radical species, TXH. and IN., it was proposed that the electron transfer is followed by proton transfer (Equation 5) during the deactivation of T_1_ state TX by indolic derivatives [8]. Therefore, the electron transfer followed by proton transfer accounts for an important deactivating pathway:

(5) $${\text{TX}}^{.-}+{\text{INH}}^{.+}\to {\text{TXH}}^{.}+{\text{IN}}^{.}$$

Moreover, there may exist another deactivating pathway which may result in the formation of TXH. and IN^.^, that is, the H-atom transfer from the quencher to T_1_ state TX as represented in the following equation:

(6) $$\text{TX}({\text{T}}_{1})+\text{INH}\to {\text{TXH}}^{.}+{\text{IN}}^{.}$$

**Scheme 1.**
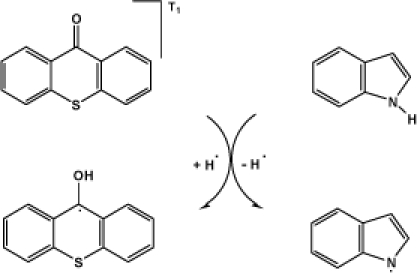
Proposed H-atom transfer-based deactivating pathway of triplet excited state thioxanthone by indole.

To explore the feasibility of this pathway, the bond dissociation enthalpy (BDE) and H-atom affinity (HAA), which have been widely employed to measure the molecular H-atom-donating and H-atom-abstracting ability, respectively [9], of TX and INH are calculated (Table 1). The BDE of N-H bond in INH is calculated to be 4.04 eV. The keto oxygen is the most favored position to accept a H-atom for TX, and the corresponding HAA is estimated as −4.56 eV. Therefore, it can be inferred that the H-atom transfer process from INH to T_1_ state TX is feasible as shown in Scheme 1. Through the H-atom transfer from INH to T_1_ state TX, TXH. and IN. are formed, and the two radical species have both been observed experimentally during the reactions of T_1_ state TX with indolic derivatives [8]. Furthermore, photoinitiated free radical polymerization is widely employed in various industrial applications [10]. TX and its derivatives are important photoinitiators, exhibiting high photoinitiation efficiency and the H-atom abstraction of T_1_ state TX from H-atom donors accounts for one important free radical generation pathway.

## 3. Theoretical Methods

The calculations were carried out using the Gaussian 03 package of programs [11] and the detailed methods are as follows. Firstly, the combined density functional theory (DFT) [12,13] method labeled as B3LYP/6-311 + G(2d,2p)//B3LYP/6-31G(d,p) was employed to estimate the electronic parameters of TX and INH [14–18], which include adiabatic electron affinity (AEA), adiabatic ionization potential (AIP), homolytic bond dissociation enthalpy (BDE) and H-atom affinity (HAA). The combined method implies that B3LYP/6-31G(d,p) was used for geometry optimization and computations of harmonic vibrational frequencies and based on the B3LYP/6-31G(d,p)-optimized geometries single-point electronic energies were obtained by B3LYP/6-311 + G(2d,2p) level in an implicit water model. For each optimized structure a frequency analysis was used to verify that it corresponds to a stationary point in the potential energy surface. The lowest triplet excitation energy (E_T1_) of TX and INH was calculated by TD-B3LYP/6-31G(d,p) method [19–21]. Solvent (water) effect was considered through the self-consistent reaction field (SCRF) method with a polarizable continuum model (PCM) [22–24] during the calculations.

## 4. Conclusions

To summarize, according to quantum chemical calculations, three postulated pathways, *i.e.,* direct electron transfer, electron transfer followed by proton transfer and direct H-atom transfer, may be involved in T_1_ state TX deactivation by INH. The present findings provide insight into the photosensitization characteristics of excited state TX.

## Figures and Tables

**Table 1.** Theoretically estimated lowest triplet excitation energy (E_T1_), adiabatic electron affinity (AEA), adiabatic ionization potential (AIP), homolytic bond dissociation enthalpy (BDE) and H-atom affinity (HAA) of thioxanthone (TX) and indole (INH) in ground (S_0_) state and triplet excited (T_1_) state in aqueous solution (in eV). 1 eV = 23.06 kcal/mol.

	**E_T1_**	**E_T1_^a^**	**AEA**	**AEA_T1_^b^**	**AIP**	**AIP_T1_^c^**	**BDE**	**HAA_T1_^d^**
**TX**	2.78	2.84 [5]	–2.77	–5.55	5.90	3.12		–4.56
**INH**	3.26	3.12 [6]	–1.21	–4.47	5.46	2.20	4.04	

a Experimental value,

b AEA_T1_ = AEA_S0_ – E_T1_;

c AIP_T1_ = AIP_S0_ – E_T1_;

d HAA_T1_ = HAA_S0_ + E_T1._
